# Retroperitoneal pneumatosis and subcutaneous emphysema after oblique lateral interbody fusion surgery: a case report

**DOI:** 10.1186/s13256-021-02980-6

**Published:** 2021-07-31

**Authors:** Chen Liu, Yu Zhang, Xin Ge, Liang Xiao, Hongguang Xu

**Affiliations:** 1 Spine Research Center of Wannan Medical College, No. 2 Zheshan West Road, Wuhu, 241001 Anhui China; 2 Department of Orthopaedics, Yijishan Hospital of Wannan Medical College, No. 2 Zheshan West Road, Wuhu, 241001 Anhui China

**Keywords:** Retroperitoneal pneumatosis, Subcutaneous emphysema, OLIF surgery, Complications, Case report, Lumbar spinal stenosis

## Abstract

**Background:**

The oblique lateral interbody fusion technique has received increasing attention for the treatment of degenerative disc disease in recent years. A series of complications have been reported, such as vascular injury, sympathetic chain injury, and transient psoas weakness, although it is regarded as a relatively safe technique.

**Case presentation:**

A 55-year-old male patient of Han nationality was diagnosed with lumbar spinal stenosis and underwent standalone oblique lateral interbody fusion surgery under general anethesia. Three days after the operation, subcutaneous gas accumulation appeared in the left lower abdomen of the patient with no inflammatory reaction of the wound. He was treated with conservative management, and the retroperitoneal pneumatosis and subcutaneous emphysema had disappeared completely 1 month later.

**Conclusions:**

To date, this is the first reported case of retroperitoneal pneumatosis and subcutaneous emphysema related to oblique lateral interbody fusion surgery, which broadens the scope of the complications of oblique lateral interbody fusion surgery.

## Background

Oblique lateral interbody fusion (OLIF) is a new technique in which operative access is gained between the abdominal aorta and the psoas major muscle [1]. It is accepted that OLIF not only avoids the excessive traction of large vessels resulting from anterior lumbar interbody fusion (ALIF) but also prevents the destruction of the psoas and lumbar plexus caused by extreme lateral interbody fusion/direct lateral interbody fusion (X/DLIF) [2]. Compared with the most popular posterior lumbar interbody fusion (PLIF) and transforaminal lumbar interbody fusion (TLIF), OLIF can prevent paraspinal muscle destruction and dural adhesion [3]. Although the incidence of complications related to this approach is relatively low, some complications have still been reported in previous studies, such as vascular injury, sympathetic chain injury, and transient psoas weakness. To the best of our knowledge, retroperitoneal pneumatosis and subcutaneous emphysema of the abdominal wall after OLIF surgery have not been reported in the literature.

We present a unique case of retroperitoneal pneumatosis and subcutaneous emphysema of the abdominal wall with a noninfectious genesis after OLIF surgery. The patient approved submission of the case for publication.

## Case presentation

**Fig. 1:**
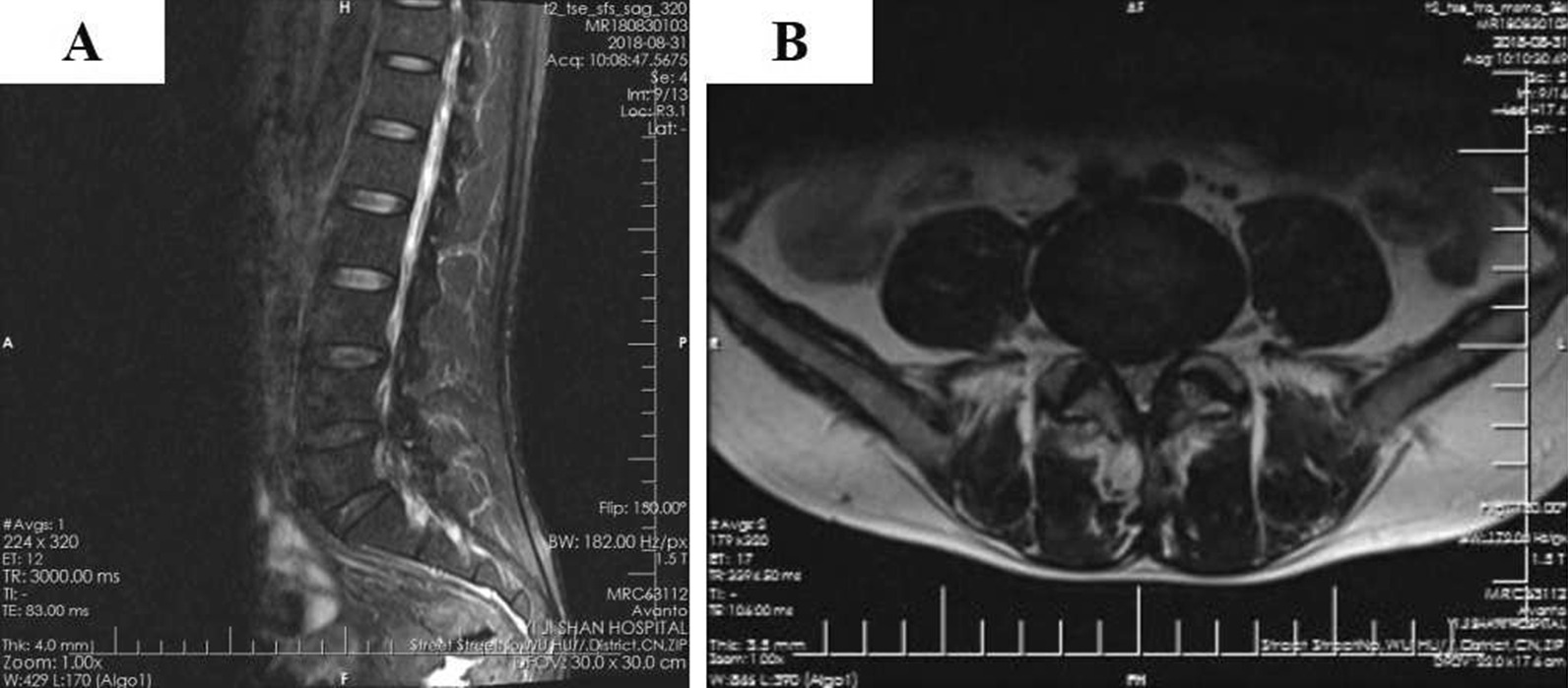
Sagittal (**A**) and transverse (**B**) fat-suppressed MRI of the lumbar spine indicating narrowing of the spinal canal at L4/5

**Fig. 2:**
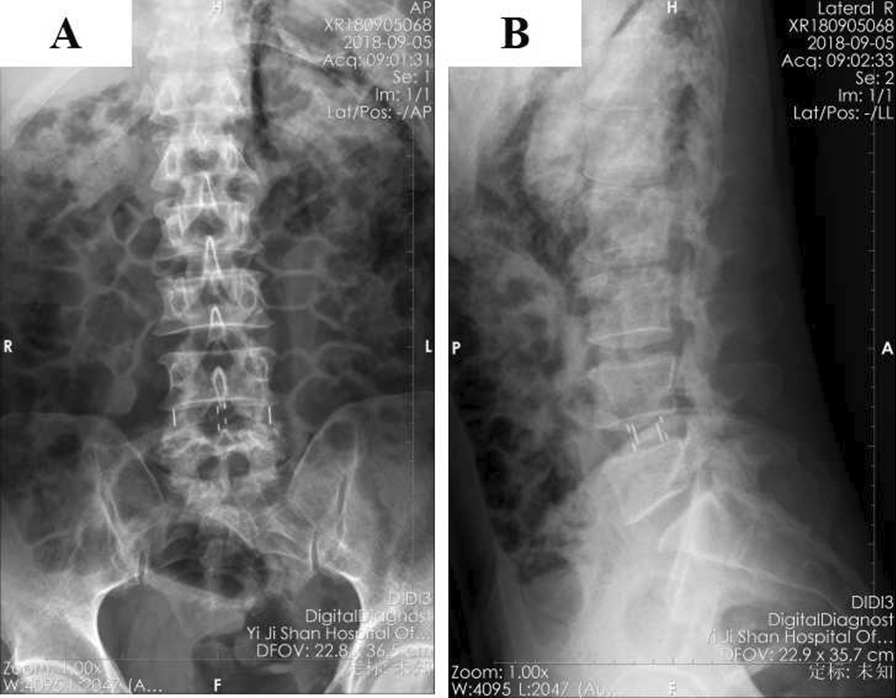
Positive (**A**) and lateral (**B**) X-rays of the lumbar spine after stand-alone OLIF surgery

**Fig. 3:**
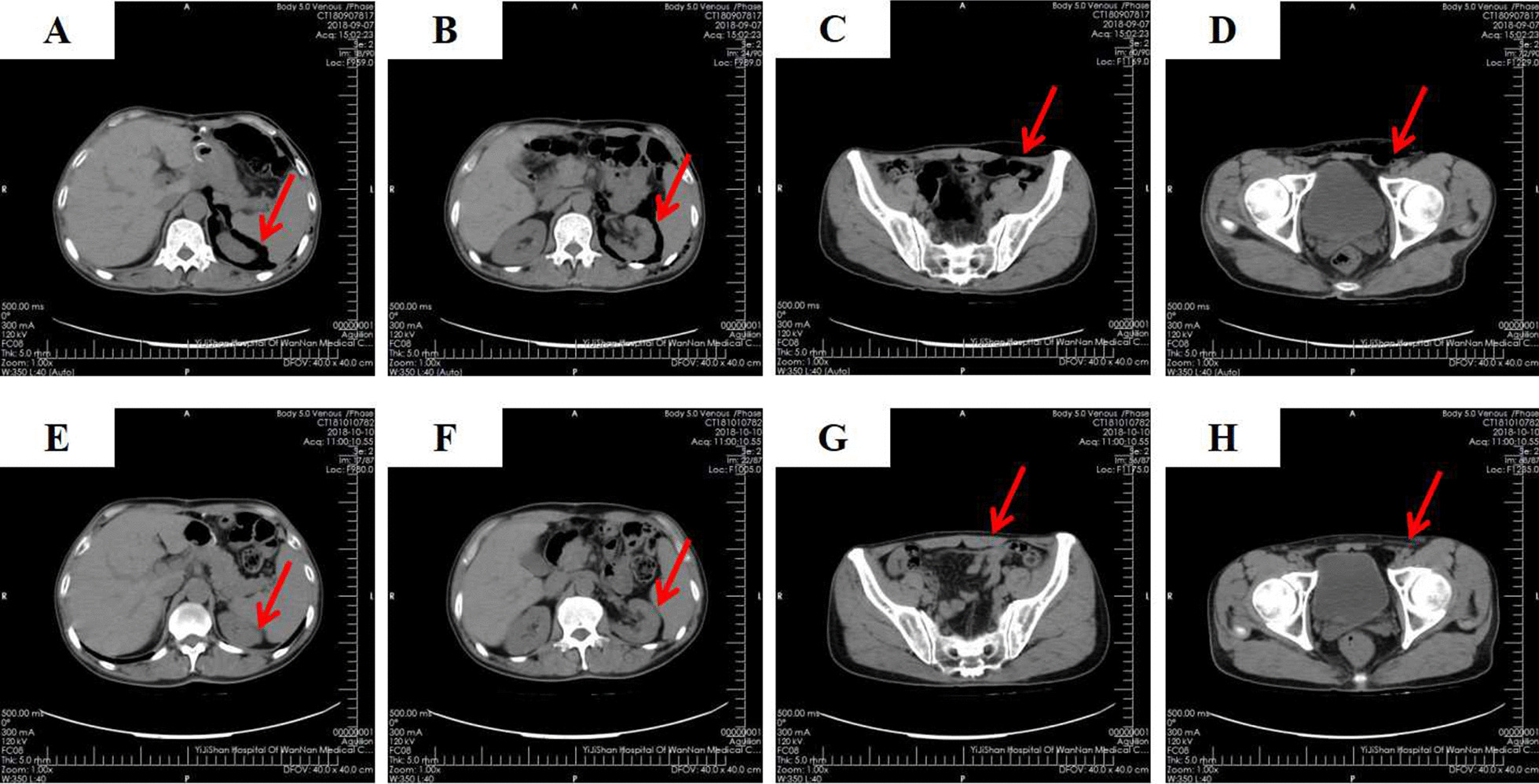
Perirenal pneumatosis **A**, **B** and subcutaneous emphysema of the abdominal wall **C**, **D** were obvious on CT 3 days after OLIF surgery (red arrow). One month later, the perirenal pneumatosis **E**, **F** and subcutaneous emphysema of the abdominal wall **G**, **H** had almost completely disappeared

A 55-year-old male patient of Han nationality was admitted to our department complaining of numbness and weakness of the lower limbs for more than 5 years and aggravated for 1 month. Lumbar magnetic resonance imaging (MRI) (Fig. 1) indicated that the L4/5 spinal canal was extremely narrow. The patient was diagnosed with lumbar spinal stenosis and underwent stand-alone OLIF surgery under general anesthesia in the right lateral decubitus position. The patient began to practice walking the day after the operation with waist protection, and the symptoms improved. Postoperative lumbar X-ray (Fig. 2) examination showed that the cage was centrally located and that the intervertebral foramen had significantly increased in size. Two days after the surgery, the patient was allowed to be discharged from the hospital with the guidance of strictly wearing waist protection and avoiding bending. Unfortunately, the patient came to our department again complaining of distention of the left lower abdomen 1 day after discharge. The wound did not appear red, and the immediate laboratory test results showed a C-reactive protein (CRP) level of 4.97 mg/L and an erythrocyte sedimentation rate (ESR) of 13.4 mm/hour. Furthermore, the patient had no fever. On physical examination, the distension of the lower left abdomen could be palpated. To clarify the abdominal condition, abdominal CT was performed. The results indicated subcutaneous gas accumulation in the left abdominal wall and retroperitoneal pneumatosis, especially around the left kidney (Fig. 3A–D). After consultation, the general surgeon recommended the application of hot compresses to the painful area. The patient was discharged from the hospital 1 week later without any discomfort. One month after the operation, the abdominal CT examination was performed again. No retroperitoneal accumulation or subcutaneous emphysema of the abdominal wall was found (Fig. 3E–H).

## Discussion

It is widely accepted that OLIF is a relatively safe technology. However, complications are inevitable. Complications can be divided into intraoperative complications and postoperative complications. As reported in previous studies, intraoperative complications include vascular injury, cage subsidence, and vertebral fractures. However, nerve injury, cage subsidence or shifting, and left lower abdominal pain are common postoperative complications [4]. To our knowledge, the current case is the first reported case of retroperitoneal pneumatosis and subcutaneous emphysema of the abdominal wall after OLIF surgery.

It is important to determine the reasons for retroperitoneal pneumatosis and subcutaneous emphysema of the abdominal wall after the operation. In our view, at least three causes should be taken into consideration. First, subcutaneous emphysema is mostly caused by serious infection from gas-forming microorganisms, as demonstrated in previous studies [5]. However, in this case, there were no signs of infection because the white blood cell count and CRP and ESR levels of the patient were normal. In addition, there was no fever, and there was no irritation or inflammation of the wound. Second, the kidney is a retroperitoneal organ. Perirenal pneumatosis mainly originates from a ruptured duodenum [6]. However, the patient showed no signs of peritoneal irritation on physical examination. Moreover, the patient showed no abnormalities in terms of diet or defecation. Finally, the most likely cause of retroperitoneal pneumatosis was air entering the retroperitoneal space during flushing of the operation area. Regarding subcutaneous emphysema of the abdominal wall, on the one hand, retroperitoneal gas could have escaped into the subcutaneous tissue of the left abdominal wall through the fascia of the external oblique abdominal muscle, which was not sutured tightly; on the other hand, premature mobilization could presumably lead to the suction of air into the wound. Julian [7] reported a case of subcutaneous emphysema after elbow arthroscopy caused by joint mobilization. The air was trapped inside and remained in the subcutaneous tissue. Much earlier, Deshmukh [8] demonstrated a case in which early knee mobilization may have led to the suction of air into the knee with every flexion and entrapment of the air in extension after knee arthroscopy. Luckily, the retroperitoneal pneumatosis and subcutaneous emphysema of the abdominal wall in this patient had disappeared 1 month after OLIF surgery without surgical or antibiotic treatment.

## Conclusions

This case highlights the implication that the surgeon should identify the causes of adverse clinical events after an operation and initiate appropriate treatment. Retroperitoneal pneumatosis and subcutaneous emphysema of the abdominal wall could be regarded as a new postoperative complication of OLIF. Patients with retroperitoneal pneumatosis and subcutaneous emphysema of the abdominal wall could be treated conservatively if they have minimal pain and are clinically stable. However, close clinical follow-up should be recommended to avoid life-threatening soft-tissue infection.

## Data Availability

Not applicable.

## Abbreviations

ALIF: Anterior lumbar interbody fusion
CT: Computed tomography
DLIF: Direct lateral interbody fusion
MRI: Magnetic resonance imaging
ODI: Oswestry Disability Index
OLIF: Oblique lateral interbody fusion
PLIF: Posterior lumbar interbody fusion
TLIF: Transforaminal lumbar interbody fusion
VAS: Visual analog scale
XLIF: Extreme lateral interbody fusion
